# Lateral or Medial Parapatellar Surgical Approach to the Valgus Osteoarthritic Knee? A Retrospective Single-Center Study

**DOI:** 10.3390/jcm11195953

**Published:** 2022-10-09

**Authors:** Piotr Dudek, Dariusz Marczak, Tomasz Okoń, Dariusz Grzelecki, Jan Szneider, Jacek Kowalczewski

**Affiliations:** 1 Centre of Postgraduate Medical Education, Department of Orthopaedics and Rheumoorthopaedics, 01-813 Warsaw, Poland; 2 Centre of Postgraduate Medical Education, Department of Orthopaedics, 01-813 Warsaw, Poland

**Keywords:** valgus knee, lateral approach, medial approach, total knee replacement

## Abstract

Aims: Total knee arthroplasty in patients with fixed valgus deformity is a demanding procedure. The aim of this study was to compare the clinical results of using the lateral approach [LA] versus the medial approach [MA] in the treatment of fixed valgus knee deformities. Methods: This single-center study compared the results of 143 consecutive patients with fixed valgus deformity (mean 21.55° valgus, mean age 68.2 years) undergoing LA Total Knee Arthroplasty [TKA] to 50 patients (mean 16.58° valgus, mean age 67.2 years) undergoing MA TKA. The mean follow-up period was 5.1 years (2–10 years). Data was collected from operative notes, routine postoperative visits, and radiological findings. Apart from a radiological evaluation, patients were clinically assessed both pre- and postoperatively using the Knee Society Score [KSS]. Descriptive statistics together with the Kolmogorov-Smirnov test, the Student’s t-test for independent samples, and the Mann-Whitney U test were used. The level of significance in this study was α = 0.05. Results: In the LA group, the KSS Knee was significantly higher than in the MA group [85.31 vs. 77.42, respectively, *p*-value < 0.001]. The difference was also in the KSS total but with no statistical significance [155.17 vs. 149.22, *p*-value 0.087]. The surgery time in the LA group was shorter than in the MA group [81 vs. 91 min, respectively, *p*-value—0.002]. The complication rate after surgery was higher in the MA group than in the LA group (14% vs. 9%, respectively). Conclusions: The lateral approach is a good alternative to the standard medial parapatellar approach in the treatment of fixed valgus knee deformities. A higher postoperative KSS Knee, shorter surgery time, and similar complication rate make the lateral approach a valuable option for treating patients with osteoarthritis and fixed valgus knee deformity.

## 1. Introduction

The valgus knee is defined as a tibiofemoral angle greater than 10° [1] Only 10 to 15% of all patients treated with total knee arthroplasty present valgus knee deformity [VKD]. Ranawat et al. describe three types of VKD with appropriate balancing techniques and implant selection according to mechanical axis deviation [2] (Table 1).

Several pathological changes may be highlighted in the VKD. The contracted structures usually include the lateral collateral ligament, the popliteus tendon, the iliotibial band [ITB], the posterolateral capsule, and the lateral patellar retinaculum [2,3,4].

In the 20th century, several modifications of the lateral approach have been described. In 1991, Keblish et al. first recommended the use of the lateral parapatellar approach in TKA with fixed valgus deformity [5]. This technique provides direct access to the lateral structures, making it easier to perform the release of contracted lateral structures, therefore ensuring proper knee balancing [3,4].

The gold standard in total knee arthroplasty is the medial parapatellar approach. It can be used in most cases. In the case of valgus knee deformities, surgical field exposure and soft tissue balance play major roles in achieving good clinical outcomes for TKA [6]. The indication for using the medial parapatellar approach is a total knee replacement [TKR] in an osteoarthritic knee joint with a varus deformity or with no deformity. The main indication for using the lateral approach is a valgus knee deformity with patella subluxation/luxation, and patella maltracking before TKR. In the authors’ institution, LA TKR in a valgus knee with a tibio-femoral angle of >10° is currently considered the “gold standard”.

The purpose of this study was to compare the surgical results and clinical and radiographic outcomes of the medial and lateral approaches for TKA in knees with fixed valgus deformities with a tibiofemoral angle greater than 10° (Grade II, III Ranawat’s Classification). There is a lot of controversy among surgeons about which approach is the most suitable, LA or MA. We hypothesized that the lateral approach would result in a similar complication risk and better outcomes than the medial parapatellar approach, with easier intraoperative knee balancing, and that it should be systematically used in most valgus knee deformities. 

## 2. Materials and Methods

### 2.1. Patient Selection

**Figure 1:**
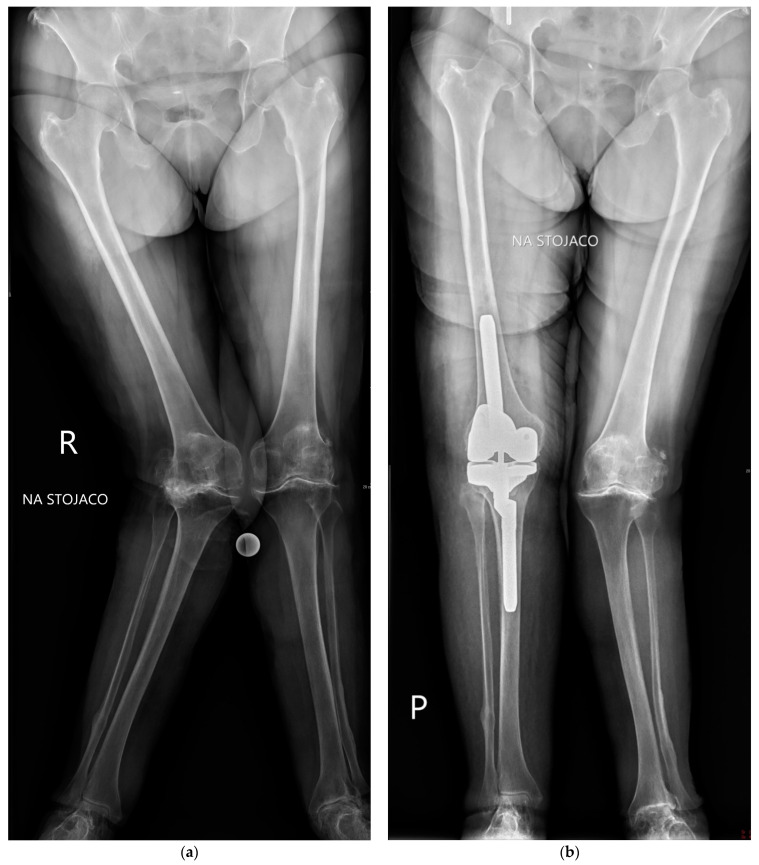
(**a**) Patient with grade III fixed valgus deformity’s long-standing X-ray. (**b**) Postoperative X-ray with mechanical axis correction.

This retrospective study consists of 193 patients who underwent surgery in the author’s institution between 2012 and 2020 due to osteoarthritis of the knee with a valgus deformity and a tibiofemoral angle of >10°. The inclusion criteria were primary osteoarthritis or osteoarthritis secondary to rheumatoid arthritis with a fixed valgus deformity at the joint level. All patients were rated as grade II or III according to Ranawat’s classification. The exclusion criteria consisted of posttraumatic osteoarthritis and a valgus deformity of the limb out of the knee joint level. According to surgical protocols, 143 patients were operated on using the lateral approach [LA] and 50 using the medial parapatellar approach [MA] to the knee joint. Data was collected from operative notes, routine postoperative visits, and radiological findings. The surgical approach was dictated mostly by the individual surgeon’s preference and experience. All patients had preoperative long-axis standing X-rays for surgery planning and postoperative long-axis standing X-rays on outpatient control visits (Figure 1a,b). Pre- and postoperative measurements were performed using CareStream software [Carestream Health, Inc., Rochester, NY, USA], while pre-operative planning was conducted in OrthoView software [Meridian Technique Limited, Hampshire, GB].

The mean follow-up period was 5.1 years (2–10 years).

### 2.2. Surgical Approach

**Figure 2:**
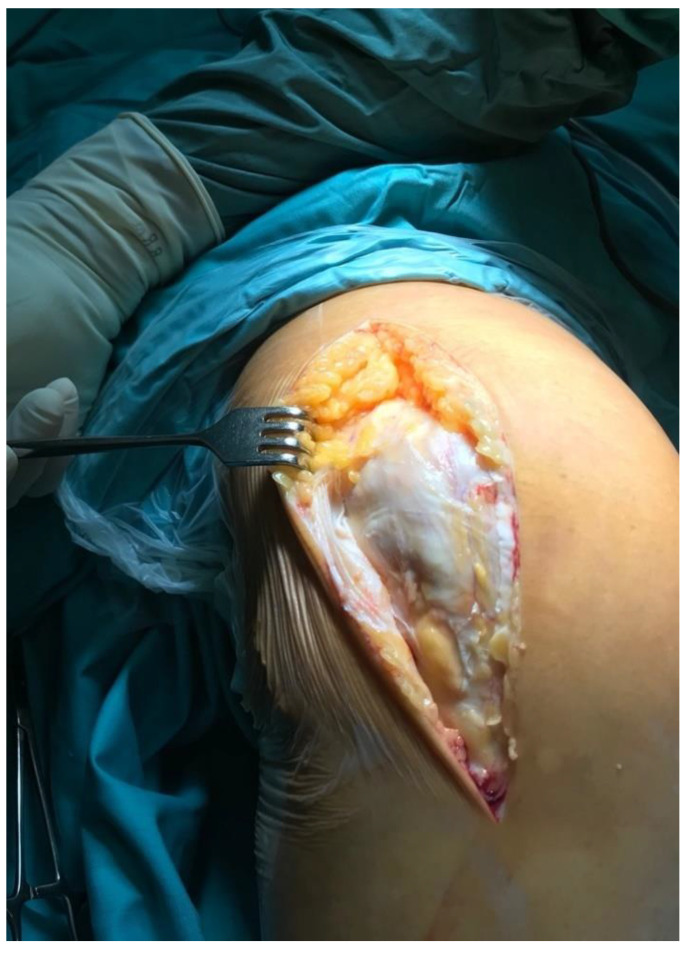
Skin incision.

**Figure 3:**
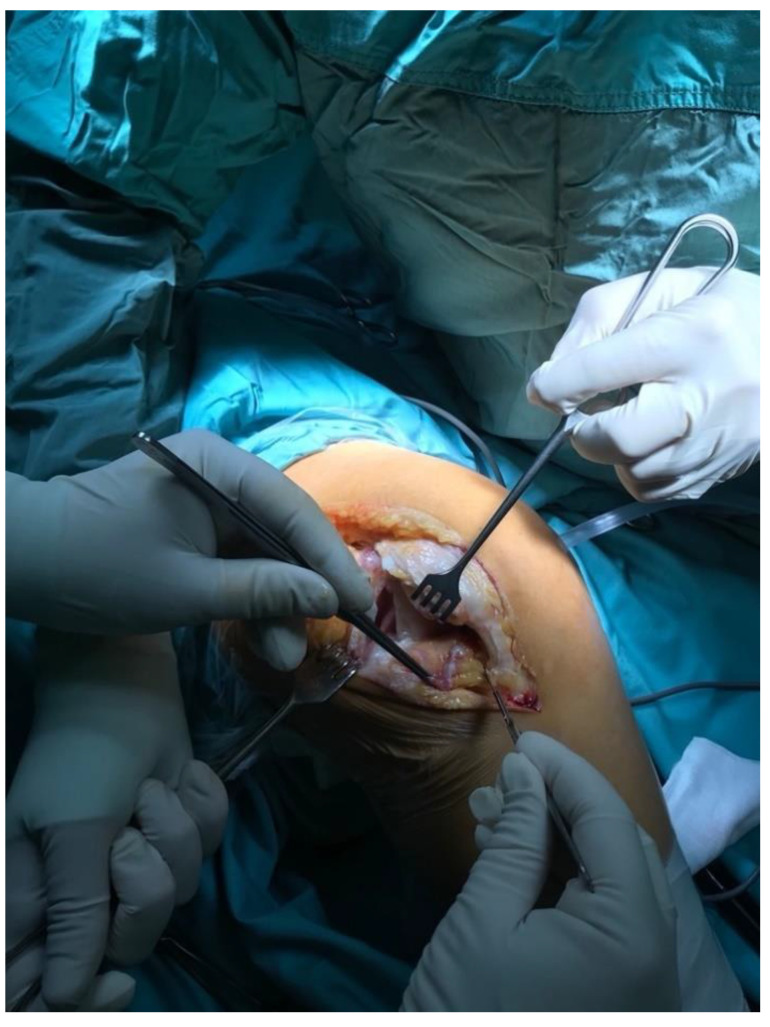
Lateral arthrotomy with the Hoffa fat pad preparation.

**Figure 4:**
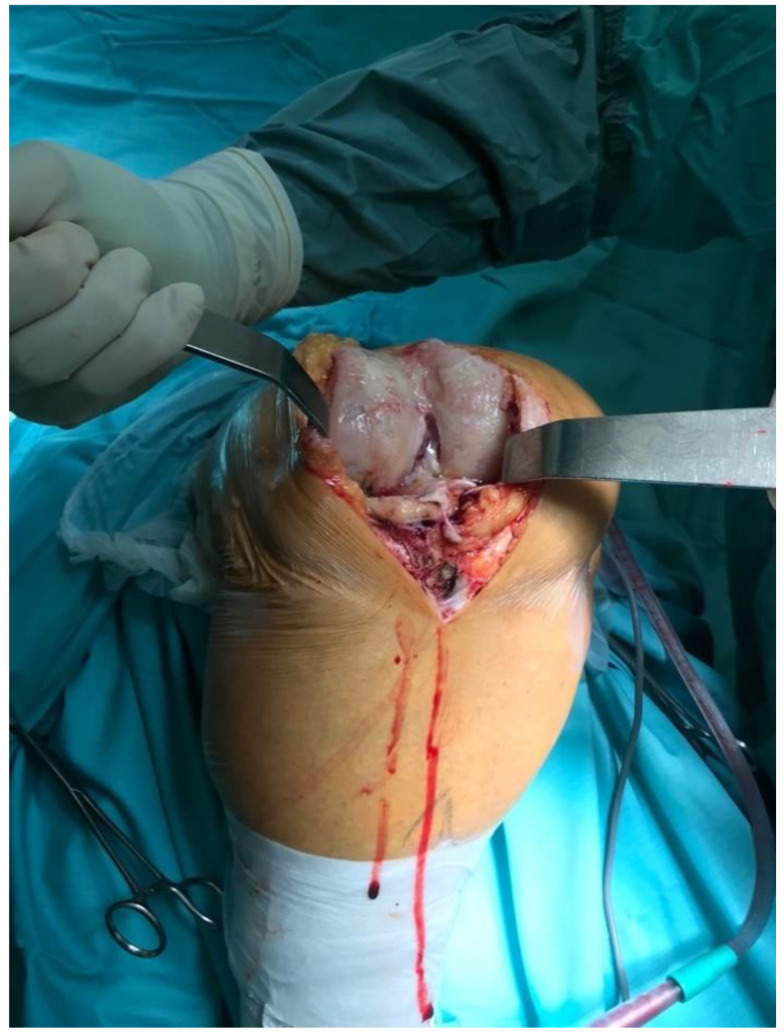
Medial displacement of the patella.

**Figure 5:**
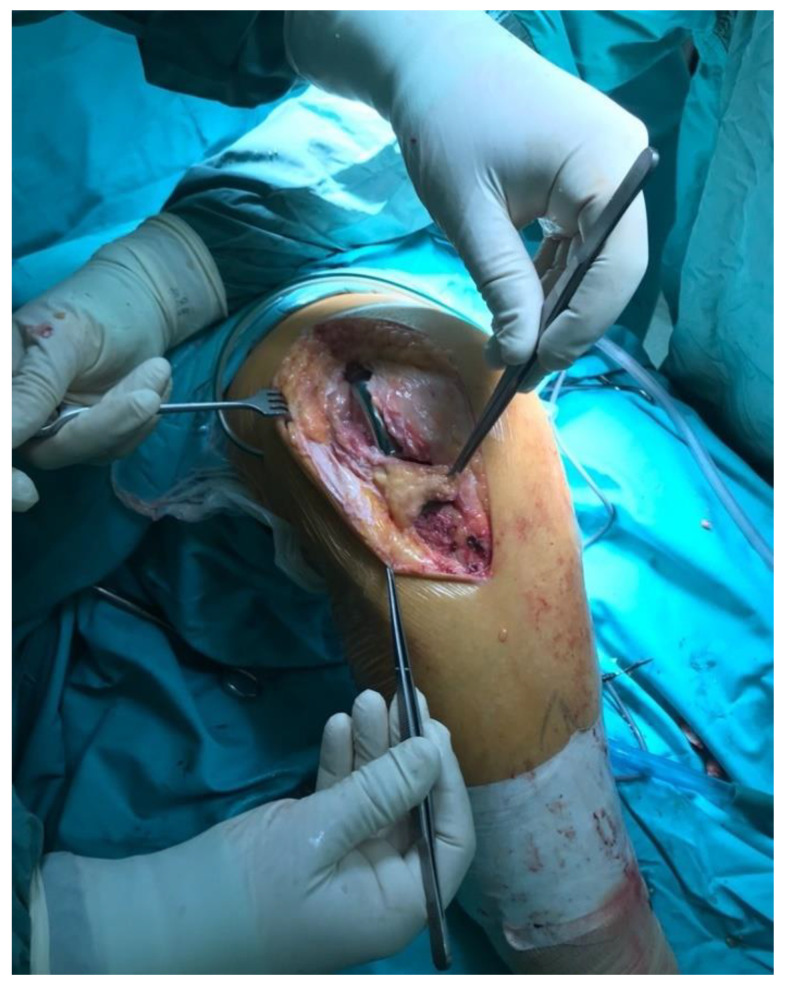
Closure technique.

**Figure 6:**
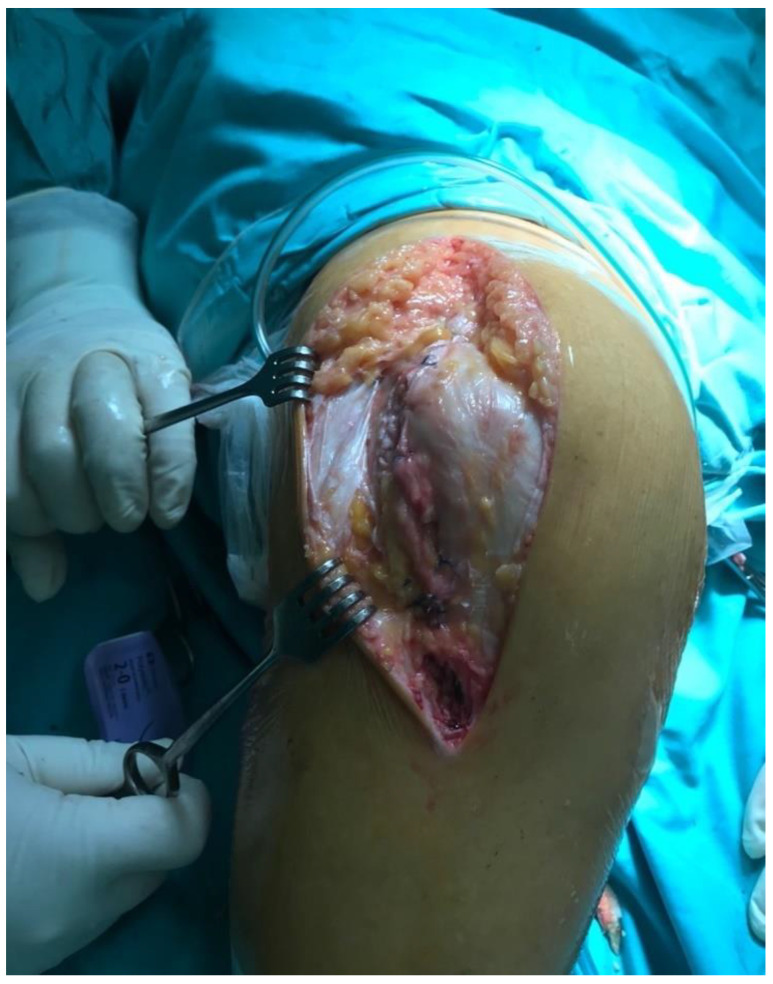
Closed joint capsule. Hoffa fat pad incorporated in the distal pole of the wound.

All knees were operated on in a tourniquet, and the standard preparation for the operated limb was performed. The first group was operated on with the use of a lateral parapatellar approach and the second with the use of a medial parapatellar approach. In the lateral approach group, a skin incision was made from the midline of the distal thigh to the lateral border of the tibial tubercle (Figure 2). Then, the lateral parapatellar arthrotomy was performed (Figure 3). The patella was dislocated medially and, if possible, everted (Figure 4). In most of the patients who were operated on using the lateral approach, eversion of the patella was impossible. Due to the shortage of tissue after the correction of the valgus deformity, the meticulous preparation of the Hoffa fat pad is essential for joint closure success (Figure 3). The lateral meniscus was preserved until the late stage of the surgery to protect the fat pad. In the next step, the ITB was released in the region of Gerdy’s tubercle, which in the authors’ opinion is obligatory in every case of valgus knee balancing. The second group consisted of patients who were operated on with the use of a standard medial parapatellar approach. In this group, all cases needed the extended lateral release of the patella during surgery due to its maltracking. In rare cases, in both groups, additional procedures to release the lateral side were needed, such as the release of the popliteus tendon [PT], pie crusting of the lateral collateral ligament [LCL], or sliding osteotomy of the lateral epicondyle. If the medial laxity was unacceptable, a constrained condylar knee [CCK] implant was preferred to a posterior-stabilized [PS] one. In severe cases, with completely unstable knees, a rotating-hinged [RH] implant was used. The closure of the joint capsule was performed in both groups with the use of a continuous monofilament loop suture. In the lateral approach group, the Hoffa fat pad was used to close the joint on the lateral side due to tissue deficit after the valgus correction (Figure 5 and Figure 6). 

### 2.3. Postoperative Assessment

As mentioned in previous paragraphs, apart from the radiological evaluation, patients were clinically assessed both pre-and postoperatively using the Knee Society Score [KSS] [7].

### 2.4. Statistical Analysis

Statistical analysis was performed with the use of IBM SPSS Statistics 27 software. Descriptive statistics were used along with the Kolmogorov-Smirnov test, the Student’s t-test for independent samples, and the Mann-Whitney U test. The level of significance in this study was α = 0.05.

The study was carried out in accordance with the World Medical Association Declaration of Helsinki and was approved by the Ethical Committee of The Centre of Postgraduate Medical Education number 50/2021.

## 3. Results

The pre-operative characteristics of the patients in both the medial and the lateral parapatellar approach groups were similar, except for the angle of deformity, which was higher in the LA group [mean 21.55° vs. 16.58°]. Additionally, the pre-operative values of the KSS were significantly lower in the LA group [KSS total 57.43 in LA vs. 79.70 in MA] (Table 2).

In the LA group, KSS Knee was significantly higher than in the MA group [85.31 vs. 77.42, respectively, *p*-value < 0.001]. The difference was also in the KSS total but with no statistical significance [155.17 vs. 149.22, *p*-value 0.087] (Table 3).

The surgery time in the LA group was shorter than in the MA group [81 vs. 91 min, respectively, *p*-value—0.002] (Table 3).

Last but not least, it is also important to note the design of the prosthesis used in particular cases. Patients operated on with the LA were more likely to receive CCK implants when compared to the MA group [29% vs. 10%, *p*-value 0.001]. In the authors’ opinion, that corresponds with a greater angle of valgus deformity in the LA group pre-operatively [21.55° vs. 16.58°, *p*-value < 0.001]. Rotating-hinge designs were used in only 2 cases—1 in the LA and 1 in the MA group; therefore, they were taken out of the analysis (Table 3).

All the data is presented in Table 2 and Table 3. 

A separate analysis was performed for patients with and without rheumatoid arthritis in the group operated on with the lateral approach. There were no statistical differences in the postoperative results for this group (Table 4).

The complication rate after surgery was higher in the MA group than in the LA group (14% vs. 9%, respectively) (Table 5). The most important difference was the occurrence of peroneal nerve palsy, which afflicted four patients in the MA group and one patient in the LA group. In all cases, the function of the peroneal nerve returned completely. Two patients from the LA group presented mild medial instability without clinical implications. Only 8 patients needed surgery due to surgical complications, 6 in the LA group (1—early, acute periprosthetic joint infection [PJI] treated with debridement, antibiotics, implant retention [DAIR], 1—aseptic loosening treated with implant exchange, 4—superficial wound-healing problems treated with skin debridement] and 2 in the MA group (1—early, acute PJI treated with DAIR, 1—healing problems treated with skin debridement).

## 4. Discussion

There is much controversy among surgeons concerning the optimal surgical approach for the treatment of the valgus knee. Decisions to use one of the two approaches described in this paper are often dictated by surgeons’ individual preferences. In the authors’ opinion, however, using the lateral approach has many important benefits. 

According to Sekiya et al., the lateral approach is beneficial in terms of knee balancing [8]. In a study conducted on 48 valgus knees [24 operated on with the medial and 24 with the lateral approach], the authors showed that using the lateral approach as part of a knee-balancing technique could reduce the rate of additional structure release such as PT or LCL. Another important issue is postoperative mean flexion, which was shown to be related to the chosen type of approach [123.8° +/−11 in LA vs. 109° +/−14.3 in MA, with *p* < 0.001]. In the authors’ study, the mean range of motion [ROM] differs in the LA and the MA, but the difference is statistically insignificant [117° vs. 115.6°, respectively].

Rawal et al. showed that similar functional results could be obtained by the LA and the MA in the treatment of the valgus knee, but in the second group of patients a higher constraint of implants was needed due to a more excessive release and joint-balancing difficulties [9]. Our study showed similar functional results for both groups, except for a higher KSS Knee in the LA group [85.31 vs. 77.42, respectively, *p*-value < 0.001].

By contrast, Gunst et al., in a retrospective study conducted on 424 knees [Ranawat Grade I deformation], stated that the results of TKA with the LA and the MA were equal [3]. These findings, however, are limited by mild valgus deformation. In addition, it may be a matter of concern that tibial tubercle osteotomy [TTO] was necessary in over 20% of all the cases of mild valgus deformity described in the study. This large number of excessive surgical approaches to the knee joint is not comparable with the authors’ experience and the material presented in this study. In the authors’ material, only one rheumatoid arthritis patient who presented a stiff knee and a severe valgus deformity needed a TTO. That patient was rated as grade III by Ranawat’s classification.

In the case of non-correctable valgus deformity, Chalidis et al. recommended the LA with tibial tubercle osteotomy [10]. The authors presented one of the biggest cohorts, including patients who underwent TKA with the LA along with TTO [57 knees]. Only three cases with complications followed this procedure, mostly in patients suffering from rheumatoid arthritis. Despite TTO giving a great visualization of the knee joint, it cannot be considered a completely safe procedure in primary TKA because of its higher risk of wound-healing problems or tibial tubercle fractures [11]. A combination of the lateral subvastus approach [LSA] with TTO is also an alternative for the MA. Hirschmann et al. proved that the LSA with TTO is statistically superior to the MA [12]. It provides a higher postoperative flexion [118° vs. 114°, respectively], lower postoperative mean VAS [0.9 vs. 1.4, respectively], and better KSS [182 vs. 171]. An extremely important fact is that patients treated with the LSA with TTO had lower medial laxity than those treated with the MA. In contrast to the study presented by Hirschmann, Hay et al. showed that there were no significant differences in a 2-year follow-up between patients who underwent TKA with the LSA with TTO and patients who were operated on with the MA [13]. The usefulness of the LA with TTO in severe valgus deformity is emphasized in the relevant literature [1,11,14]. In severe valgus knee TKA, this approach provides great conditions to perform the proper balancing of lateral contracted structures as it is a part of this approach. In addition, if needed, it allows for tibial tubercle medialization in cases of patella maltracking. 

Patella devascularization is always a risk when using the standard MA with excessive lateral release [15,16,17]. The lateral approach does not need any additional patella release procedures in the valgus knee, and therefore it does impact the blood supply to the patella and does not cause a higher risk of fracture or necrosis. This is usually caused due to jeopardizing the lateral superior genicular artery.

A recent publication by Greenberg et al. revealed many advantages of the LA over the MA [18]. Applying the LA to TKA in a valgus knee resulted in a shortening of the mean surgery time [137 min. in MA to 87 min. in LA] and in using less constrained prostheses [16% in LA vs. 9% in MA]. The study also showed a trend of a better mechanical axis correction in patients who underwent TKA with the LA. The authors’ study confirms most of these advantages, except for a number of constrained implants used in the LA group. A systematic review by Wang et al. suggests that the LA with TTO or a quadriceps muscle snip is more effective than the MA in fixed valgus deformities. The authors emphasize the easier release of lateral structures, better knee joint visualization, and better patella tracking after TKA, but the requirement of a long learning curve is a clear disadvantage [19]. A meta-analysis by Xu et al. showed that current data suggest that the LA in the treatment of a valgus knee is superior to the MA in terms of the KSS but similar in operative time, blood loss, WOMAC, ROM, pain, valgus control, and total complications [6]. In the authors’ study, only KSS Knee showed higher results in the LA group than in the MA group [85.31 vs. 77.42, respectively, *p*-value < 0.001]. Another difference was a shorter surgery time for the LA when compared to the MA [81 min. vs. 91.4, *p*-value-0.002].

### Study Limitations

The authors are aware of the weak points of this study, such as unequal study groups with larger deformities seen in the LA group. Nevertheless, the clinical results of the LA group treatment seem to be equal or superior to the MA group, which supports the thesis that the lateral approach to a valgus osteoarthritic knee is superior to the medial parapatellar one. There is also a potential selection bias since the surgical approach was dictated mostly by the individual surgeon’s preference and experience.

## 5. Conclusions

In conclusion, the lateral approach in total knee arthroplasty is a good alternative to the standard medial parapatellar approach in fixed valgus knee deformity. A higher postoperative KSS Knee, shorter surgery time, and similar complication rate make the lateral approach a valuable option for treating patients with osteoarthritis and valgus knee deformity. 

## Figures and Tables

**Table 1:** Ranawat’s classification of the valgus knee.

Type	I	II	III
Correction	Full	No	No
Grade of deformity	Minimal	Substantial	Severe osseus
Medial Collateral Ligament	Functional and intact	Elongated, functional	severely impaired
Angle of deformity	<10	10–20	>20
Implant type	Posterior-Stabilized [PS]	PS/Constrained Condylar Knee [CCK]	CCK/Hinged

**Table 2:** Patients’ demographic and pre-operative data.

	Lateral Approach [LA] (*n* = 143)		Medial Approach [MA] (*n* = 50)				95% CI		
	M	SD	M	SD	t	*p*	LL	UL	d Cohen’s
Age at the time of surgery (years)	68.2	11.2	67.2	9.4	0.55	0.583	−2.52	4.47	0.09
Woman (%)	0.9	0.3	0.8	0.4	0.94	0.348	−0.06	0.17	0.17
Pre-op Range of Motion [ROM] (°)	101.3	20.9	102.9	25.2	−0.41	0.680	−9.54	6.25	0.07
Body Mass Index [BMI] (kg/m^2^)	28.5	5.1	28.0	4.2	0.68	0.497	−1.03	2.12	0.11
Pre-op Knee Society Score [KSS] Knee	27.8	15.7	37.9	17.3	−3.82	<0.001	−15.35	−4.90	0.63
Pre-op KSS Function	29.6	20.3	41.8	16.7	−4.18	<0.001	−17.91	−6.38	0.62
Pre-op KSS Total	57.4	32.9	79.7	29.4	−4.24	<0.001	−32.64	−11.90	0.70
Pre-op subluxated patella (%)	0.3	0.5	0.4	0.5	−0.91	0.364	−0.23	0.08	0.15
Pre-op valgus (°)	21.6	7.2	16.6	6.3	4.31	<0.001	2.69	7.24	0.71

**Table 3:** Comparison of postoperative variables in medial and lateral approaches.

	LA (*n* = 143)		MA (*n* = 50)				95% CI		
	M	SD	M	SD	t	*p*	LL	UL	d Cohen’s
Post-op ROM (°)	117	13.6	115.6	13.3	0.66	0.51	−2.91	5.84	0.11
Post-op KSS Knee	85.3	9.7	77.4	14.4	3.61	<0.001	3.53	12.25	0.71
Post-op KSS Function	69.9	15.0	71.8	14.7	−0.79	0.431	−6.79	2.91	0.13
Post-op KSS Total	155.2	19.5	149.2	25.0	1.72	0.087	−0.88	12.77	0.28
PS designs	0.7	0.5	0.9	0.3	−3.00	0.003	−0.30	−0.06	0.42
CCK designs	0.3	0.5	0.1	0.3	3.37	0.001	0.08	0.31	0.46
Surgery time (min.)	81.0	11.8	91.4	21.2	−3.26	0.002	−16.65	−4.00	0.70
Post-op valgus (°)	6.0	2.9	5.2	2.3	1.71	0.089	−0.12	1.68	0.28
Angle of correction (°)	15.6	7.5	11.4	6.8	3.52	0.001	1.87	6.63	0.58

**Table 4:** Rheumatoid [RA] vs. non-rheumatoid [Non-RA] patients in lateral approach.

	Non-RA (*n* = 107)		RA (*n* = 36)				
	M	Me	M	Me	Z	*p*	η^2^
Age at the time of surgery (years)	70.6	71.0	60.9	64.0	−4.30	<0.001	0.13
Woman (%)	0.9	1.0	0.9	1.0	−0.49	0.627	0.00
Pre-op ROM (°)	102.1	105.0	98.8	105.0	−0.64	0.521	0.00
Post-op ROM (°)	116.4	120.0	119.0	120.0	−0.69	0.491	0.00
BMI (kg/m^2^)	29.2	28.7	26.7	24.9	−2.67	0.008	0.05
Pre-op KSS Knee	27.9	29.0	27.6	27.5	−0.05	0.957	0.00
Pre-op KSS Function	29.4	30.0	30.1	37.5	−0.35	0.729	0.00
Pre-op KSS Total	57.3	65.0	57.7	66.0	−0.15	0.882	0.00
Post-op KSS Knee	85.3	88.0	85.3	88.0	−0.60	0.551	0.00
Post-op KSS Function	70.5	70.0	68.1	70.0	−0.93	0.355	0.01
Post-op KSS Total	155.8	157.0	153.4	157.5	−0.45	0.652	0.00
PS designs	0.7	1.0	0.8	1.0	−1.60	0.109	0.02
CCK designs	0.3	0.0	0.2	0.0	−1.51	0.132	0.02
Pre-op subluxated patella (%)	0.4	0.0	0.3	0.0	−1.16	0.247	0.01
Surgery time (min.)	81.3	80.0	80.4	77.0	−0.74	0.461	0.00
Pre-op valgus (°)	21.9	21.0	20.5	20.0	−0.98	0.328	0.01
Post-op valgus (°)	5.8	6.0	6.6	6.0	−1.27	0.204	0.01
Angle of correction (°)	16.2	15.0	13.9	13.0	−1.44	0.151	0.01

**Table 5:** Complications.

Complication	Lateral Approach (No./%)	Medial Approach (No./%)
Peroneal nerve palsy	1/0.7%	4/8%
Acute, early Periprosthetic Joint Infection [PJI]	1/0.7%	1/2%
Wound-healing problems	4/2.8%	1/2%
Persistent patella dislocation	1/0.7%	1/2%
Medial instability	2/1.4%	-
Aseptic loosening	1/0.7%	-
Total	10/9%	7/14%

## Data Availability

Not applicable.
